# An Overview of Image Caption Generation Methods

**DOI:** 10.1155/2020/3062706

**Published:** 2020-01-09

**Authors:** Haoran Wang, Yue Zhang, Xiaosheng Yu

**Affiliations:** ^1^College of Information Science and Engineering, Northeastern University, China; ^2^Faculty of Robot Science and Engineering, Northeastern University, China

## Abstract

In recent years, with the rapid development of artificial intelligence, image caption has gradually attracted the attention of many researchers in the field of artificial intelligence and has become an interesting and arduous task. Image caption, automatically generating natural language descriptions according to the content observed in an image, is an important part of scene understanding, which combines the knowledge of computer vision and natural language processing. The application of image caption is extensive and significant, for example, the realization of human-computer interaction. This paper summarizes the related methods and focuses on the attention mechanism, which plays an important role in computer vision and is recently widely used in image caption generation tasks. Furthermore, the advantages and the shortcomings of these methods are discussed, providing the commonly used datasets and evaluation criteria in this field. Finally, this paper highlights some open challenges in the image caption task.

## 1. Introduction

The development of the image description system may help the visually impaired people “see” the world in the future. Recently, it has drawn increasing attention and become one of the most important topics in computer vision [1–11]. Early image description generation methods aggregate image information using static object class libraries in the image and modeled using statistical language models. Aker and Gaizauskas [12] use a dependency model to summarize multiple web documents containing information related to image locations and propose a method for automatically tagging geotagged images. Li et al. [13] propose a n-gram method based on network scale, collecting candidate phrases and merging them to form sentences describing images from zero. Yang et al. [14] propose a language model trained from the English Gigaword corpus to obtain the estimation of motion in the image and the probability of colocated nouns, scenes, and prepositions and use these estimates as parameters of the hidden Markov model. The image description is obtained by predicting the most likely nouns, verbs, scenes, and prepositions that make up the sentence. Kulkarni et al. [15] propose using a detector to detect objects in an image, classifying each candidate region and processing it by a prepositional relationship function and finally applying a conditional random field (CRF) prediction image tag to generate a natural language description. Object detection is also performed on images. Lin et al. [16] used a 3D visual analysis system to infer objects, attributes, and relationships in an image and convert them into a series of semantic trees and then learn the grammar to generate text descriptions for these trees.

Some indirect methods have also been proposed for dealing with image description problems, such as the query expansion method proposed by Yagcioglu et al. [17], by retrieving similar images from a large dataset and using the distribution described in association with the retrieved images. The expression is used to create an extended query, and then the candidate descriptions are reordered by estimating the cosine between the distributed representation and the extended query vector, and finally, the closest description is taken as a description of the input image. In summary, the methods described are brainstorming and have their own characteristics, but all have the common disadvantage that they do not make intuitive feature observations on objects or actions in the image, nor do they give an end-to-end mature general model to solve this problem. The efficiency and popularization of neural networks have made breakthroughs in the field of image description and saw new hopes until the advent of the era of big data and the outbreak of deep learning methods.

In this paper, we review the development process of image description methods in recent years and summarize the basic framework and some improved methods. Then, we analyze the advantages and shortcomings of existing models and compare their results on public large-scale datasets. Finally, we summarize some open challenges in this task.

This paper is organized as follows. The second part details the basic models and methods. The third part focuses on the introduction of attention mechanism to optimize the model and make up for the shortcomings. The fourth part introduces the common datasets come up by the image caption and compares the results on different models. Different evaluation methods are discussed. The fifth part summarizes the existing work and proposes the direction and expectations of future work.

## 2. Feature Extraction Methods

Image caption models can be divided into two main categories: a method based on a statistical probability language model to generate handcraft features and a neural network model based on an encoder-decoder language model to extract deep features. The specific details of the two models will be discussed separately.

### 2.1. Handcraft Features with Statistical Language Model

**Figure 1:**
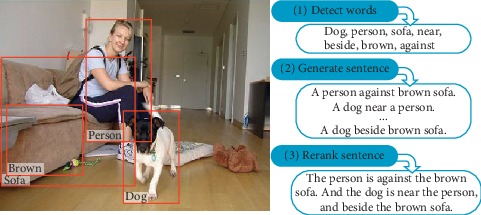
Method based on the visual detector and language model.

This method is a Midge system based on maximum likelihood estimation, which directly learns the visual detector and language model from the image description dataset, as shown in Figure 1. Fang et al. [18] first analyze the image, detect the object, and then generate a caption. Words are detected by applying a convolutional neural network (CNN) to the image area [19] and integrating the information with MIL [20]. The structure of the sentence is then trained directly from the caption to minimize the priori assumptions about the sentence structure. Finally, it turns an image caption generation problem into an optimization problem and searches for the most likely sentence.

The implementation steps are as follows:

1. Detect a set of words that may be part of the image caption. We detect the words from the given vocabulary according to the content of the corresponding image based on the weak monitoring method in multi-instance learning (MIL) in order to train the detectors iteratively.
2. Running a fully convolutional network on an image, we get a rough spatial response graph. Each position in the response map corresponds to a response obtained by applying the original CNN to the region of the input image where the shift is shifted (thus effectively scanning different locations in the image to find possible objects). By upsampling the image, we get a response map on the final fully connected layer and then implement the noisy-OR version of MIL on the response map for each image. Each word produces a single probability.
3. The process of caption generation is searching for the most likely sentence under the condition of the visually detected word set. The language model is at the heart of this process because it defines the probability distribution of a sequence of words. Although the maximum entropy language model (ME) is a statistical model, it can encode very meaningful information. For example, “running” is more likely to follow the word “horse” than “speaking.” This information can help identify the wrong words and encode commonsense knowledge.
4. There are similar ways to use the combination of attribute detectors and language models to process image caption generation. Devlin et al. [21] used a combination of CNN and k-NN methods and a combination of a maximum entropy model and RNN to process image description generation tasks. Kenneth Tran proposed an image description system, [22] using CNN as a visual model to detect a wide range of visual concepts, landmarks, celebrities, and other entities into the language model, and the output results are the same as those extracted by CNN. The vectors together are used as input to the multichannel depth-similar model to generate a description.

### 2.2. Deep Learning Features with Neural Network

The recurrent neural network (RNN) [23] has attracted a lot of attention in the field of deep learning. It was originally widely used in the field of natural language processing and achieved good results in language modeling [24]. In the field of speech, RNN converts text and speech to each other [25–31], machine translation [32–37], question and answer session [38–43], and so on. Of course, they are also used as powerful language models at the level of characters and words. Currently, word-level models seem to be better than character-level models, but this is certainly temporary. RNN is also rapidly gaining popularity in computer vision. For example, frame-level video classification [44–46], sequence modeling [47, 48], and recent visual question-answer tasks.

**Figure 2:**
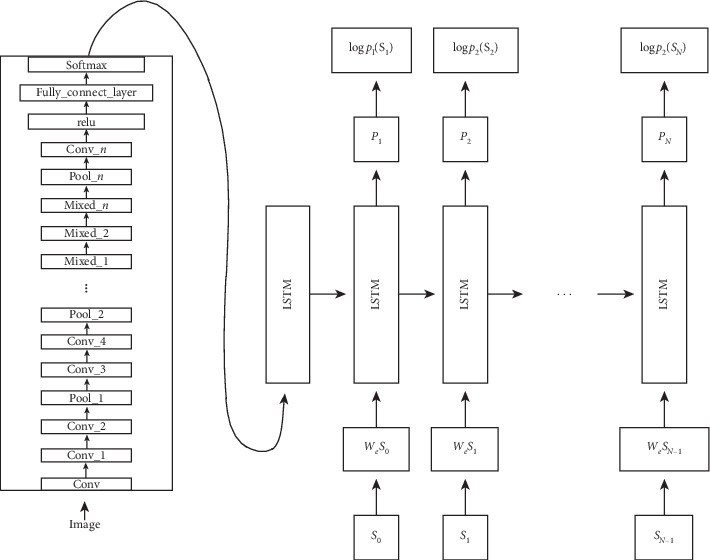
Model based on encoder-decoder.

**Figure 3:**
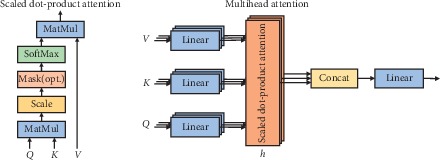
(a) Scaled dot-product attention. (b) Multihead attention.

As shown in Figure 2, the image description generation method based on the encoder-decoder model is proposed with the rise and widespread application of the recurrent neural network [49]. In the model, the encoder is a convolutional neural network, and the features of the last fully connected layer or convolutional layer are extracted as features of the image. The decoder is a recurrent neural network, which is mainly used for image description generation. Because RNN training is difficult [50], and there is a general problem of gradient descent, although it can be slightly compensated by regularization [51], RNN still has a fatal flaw that it can only remember the contents of the previous limited time unit, and LSTM [52] is a special RNN architecture that can solve problems such as gradient disappearance, and it has long-term memory. In recent years, the LSTM network has performed well in dealing with video-related context [53–55]. Similar with video context, the LSTM model structure in Figure 3 is generally used in the text context decoding stage.

## 3. Attention Mechanism

Attention mechanism, stemming from the study of human vision, is a complex cognitive ability that human beings have in cognitive neurology. When people receive information, they can consciously ignore some of the main information while ignoring other secondary information. This ability of self-selection is called attention. This mechanism was first proposed to be applied to the image classification in the field of visual images using the attention mechanism on the RNN model [56]. In natural language processing, when people read long texts, human attention is focused on keywords, events, or entities. A large number of experiments have proved that the attention mechanism is applied in text processing, for example, machine translation [35, 57], abstract generation [58, 59], text understanding [60–63], text classification [64–66], visual captioning [67, 68], and other issues, the results achieved remarkable, and the following describes the application of different attention mechanism methods in the image description basic framework introduced in the second part, so that its effect is improved.

In neural network models, the realization of the attention mechanism is that it allows the neural network to have the ability to focus on its subset of inputs (or features)—to select specific inputs or features. The main part of the attention mechanism is the following two aspects: the decision needs to pay attention to which part of the input; the allocation of limited information processing resources to the important part. At present, the mainstream attention mechanism calculation formulas are shown in equations (1) and (2); the design idea is to link the target module *m*_*t*_ with the source module *m*_*s*_ through a function and finally normalize to obtain the probability distribution:

(1) $$\begin{array}{c} a_{t}=\text{align}\left(m_{t},m_{s}\right)=\frac{\exp\left(f\left(m_{t},m_{s}\right)\right)}{\sum_{s}\exp\left(f\left(m_{t},m_{s^{\prime }}\right)\right)}, \end{array}$$

(2) $$\begin{array}{c} f\left(m_{t},m_{s}\right)=\left\{\begin{array}{cc} m_{t}^{T}m_{s}, & \text{dot,}\\ m_{t}^{T}W_{a}m_{s}, & \text{general,}\\ W_{a}\left[m_{t};m_{s}\right], & \text{concat,}\\ v_{a}^{T}\tanh\left(W_{a}m_{t}+U_{a}m_{s}\right), & \text{perception.} \end{array}\right. \end{array}$$

Based on the advantages of the attention mechanism mentioned above, this chapter details the various achievements of the attention mechanism algorithm and its application in image description generation.

### 3.1. Soft Attention

Dzmitry et al. [57] first proposed the soft attention model and applied it to machine translation. In fact, “soft” refers to the probability distribution of attention distribution. For any word in the input sentence *S*, the probability is given according to the context vector *Z*_*t*_ [69]. Finally, the weighted sum of all regions is calculated to get the probability distribution:

(3) $$\begin{array}{c} E_{p\left(s_{t}\left|a\right.\right)}\left[{\hat{z}}_{t}\right]=\overset{L}{\underset{i=1}{\sum }}\alpha_{t,i}a_{i}. \end{array}$$

A deterministic attention model is formulated by computing a soft attention weighted attention vector [57]:

(4) $$\begin{array}{c} \Phi \left(\left\{a_{i}\right\},\left\{\alpha_{i}\right\}\right)=\overset{L}{\underset{i}{\sum }}\alpha_{i}a_{i}. \end{array}$$

The objective function can be written as follows:

(5) $$\begin{array}{c} L=-\log\left(P\left(y\left|x\right.\right)\right)+\lambda \overset{L}{\underset{i}{\sum }}{\left(1-\overset{C}{\underset{t}{\sum }}\alpha_{t,i}\right)}^{2}. \end{array}$$

Soft attention is parameterized and therefore can be embedded and modeled for direct training. Gradient can be passed back through the attention mechanism module to other parts of the model.

### 3.2. Hard Attention

Unlike the soft attention mechanism, which focuses on calculating the weighted sum of all regions, hard attention only focuses on one location and is a process of randomly selecting a unique location. It samples the hidden state of the input by probability, rather than the hidden state of the entire encoder. The context vector *Z*_*t*_ [69] is calculated as follows:

(6) $$\begin{array}{c} p\left(s_{t,i}=1\left|a\right.\right)=\alpha_{t,i},\\ {\hat{z}}_{t}=\overset{L}{\underset{i=1}{\sum }}s_{t,i}a_{i}, \end{array}$$

where *s*_*t*,*i*_ refers to whether to select the *i*-th position in the *L* feature maps, if selected, set to 1, otherwise the opposite.

In order to achieve gradient backpropagation, Monte Carlo sampling is needed to estimate the gradient of the module. One disadvantage of hard attention is that information is selected based on the method of maximum sampling or random sampling. Therefore, the functional relationship between the final loss function and the attention distribution is not achievable, and training in the backpropagation algorithm cannot be used.

### 3.3. Multihead Attention

In general, we can represent input information in a key-value pair format, where “key” is used to calculate the attention distribution and “value” is used to generate the selected information. The multiheaded attention mechanism uses a plurality of keys, values, and queries to calculate a plurality of information selected from the input information in parallel for linear projection. As shown in Figure 3, each attention focuses on different parts of the input information to generate output values, and finally, these output values are concatenated and projected again to produce the final value [70]:

(7) $$\begin{array}{c} \text{MultiHead}\left(Q,K,V\right)=\text{Concate}\left({\text{head}}_{1},\ldots ,{\text{head}}_{h}\right)W^{O},\\ {\text{wherehead}}_{i}=\text{Attention}\left(QW_{i}^{Q},KW_{i}^{K},VW_{i}^{V}\right). \end{array}$$

### 3.4. Scaled Dot-Product Attention

Scaled dot-product attention [70] performs a single attention function using keys, values, and query matrices:

(8) $$\begin{array}{c} \text{Attention}\left(Q,K,V\right)=\text{soft }\max\left(\frac{QK^{T}}{\sqrt{d_{k}}}\right)V. \end{array}$$

Additional attention is paid to the compatibility function using a feedforward network with a single hidden layer. In practice, the scaled-down dot product is faster and more space-efficient than the multiheaded attention mechanism because it can be implemented using a highly optimized matrix multiplication code.

### 3.5. Global Attention

**Figure 4:**
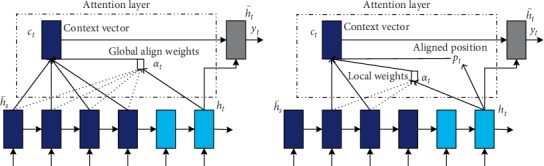
(a) Global attention model and (b) local attention model.

The main idea of global attention [71] is to consider the hidden layer state of all encoders. It obtains the attention weight distribution by comparing the current decoder hidden layer state with the state of each encoder hidden layer. It is similar to soft; that is, in the process of decoding, each time step needs to calculate the attention weight of each word in the encoding and then weights the context vector. The overall flow is shown in Figure 4. Since it chooses to focus on all the encoder inputs when calculating each decoder state, the amount of calculation is relatively large.

### 3.6. Local Attention

Local attention [71] first finds an alignment position and then calculates the attention weight in the left and right windows where its position is located and finally weights the context vector. This is actually a mixed compromise between soft and hard. The main advantage of local attention is to reduce the cost of the attention mechanism calculation. In the calculation, the local attention is not to consider all the words on the source language side, but to predict the position of the source language end to be aligned at the current decoding according to a prediction function and then navigate through the context window, considering only the words within the window.

### 3.7. Adaptive Attention with Visual Sentinel

**Figure 5:**
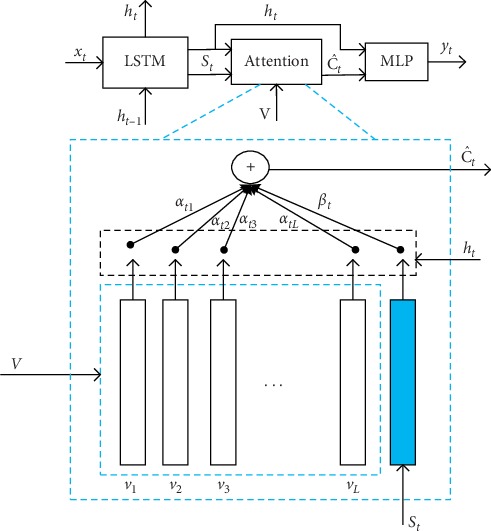
Adaptive attention model with visual sentinel.

For most of the attention models used for image caption and visual question and answer, regardless of which word is generated next, the image is focused on in each time step [72–74]. However, not all words have corresponding visual signals. The adaptive attention mechanism and the visual sentinel [75] solve the problem of when to add attention mechanisms and where to add them in order to extract meaningful information for sequence words. As shown in Figure 5, the context vector is considered to be the residual visual information of the LSTM hidden state. It reduces the uncertainty and supplements the informational of the next word prediction in the current hidden state. The calculation is as follows:

(9) $$\begin{array}{c} Ct=g\left(V,ht\right)=\overset{k}{\underset{i=1}{\sum }}\alpha_{ti}v_{ti}=\text{soft }\max\left(z_{t}\right)\cdot v_{ti}=\text{soft }\max\left(w_{h}^{T}\tanh\left(W_{V}V+\left(W_{g}h_{t}\right)I^{T}\right)\right)\cdot v_{ti},\\ {\hat{c}}_{t}=\beta_{t}s_{t}+\left(1-\beta_{t}\right)c_{t}, \end{array}$$

where the adaptive context vector is defined as ${\hat{c}}_{t}$, which is modeled as a mixture of spatial image features (i.e., the context vector of the spatial attention model) and the visual sentinel vector *β*_*t*_. It determines how much new information the network takes into account from the image and what it already knows in decoding the memory.

### 3.8. Semantic Attention

Semantic attention [76] selectively handles semantic concepts and fuses them into the hidden state and output of LSTM. Selection and fusion form feedback that connects top-down and bottom-up calculations. First, multiple top attribute and bottom-up features are extracted from the input image using multiple attribute detectors (AttrDet), and then all visual features are input as attention weight to a recurrent neural network (RNN) input and state calculation. The implementation is as follows:

(10) $$\begin{array}{c} x_{0}=\Phi_{0}\left(v\right)=W^{x,v}v,\\ h_{t}=\text{RNN}\left(h_{t-1},x_{t}\right),\\ Y_{t}∼p_{t}=\varphi \left(h_{t},\left\{A_{i}\right\}\right),\\ x_{t}=\phi \left(Y_{t-1},\left\{A_{i}\right\}\right),\;t>0, \end{array}$$

**Figure 6:**
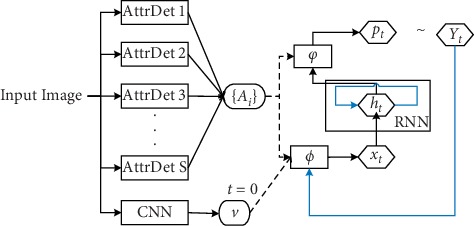
Semantic attention.

The entire model architecture is shown in Figure 6.

### 3.9. Spatial and Channel-Wise Attention

**Figure 7:**
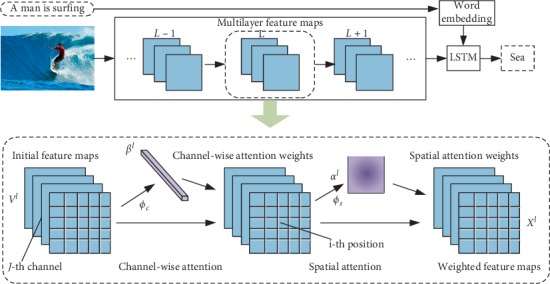
SCA-CNN model.

Spatial and channel attention [77] is the process of selecting semantic attributes according to the needs of the sentence context as shown in Figure 7. It uses the attention mechanism according to the extracted semantics in the encoding process, in order to overcome the general attention mechanism in decoding. Pay attention to the problem of overrange when using the last layer of the process. For example, when we want to predict “cake,” channel-wise attention (e.g., in the “convolution 5_3/convolution 5_4 feature map”) will be based on “cake,” “fire,” “light,” and “candle” and equivalent shape semantics, and more weight is assigned on the channel. Secondly, since the feature map depends on its underlying feature extraction, it is natural to apply attention in multiple layers; this allows obtaining visual attention on multiple semantic abstractions.

### 3.10. Areas of Attention

**Figure 8:**
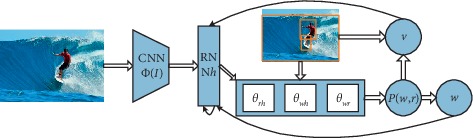
Areas of attention.

Pedersoli et al. [4] proposed a note-taking model (Figure 8). The method uses three pairs of interactions to implement an attention mechanism to model the dependencies between the image region, the title words, and the state of the RNN language model. Compared with the previous method of associating only the image region with the RNN state, this method allows a direct association between the title word and the image region, not only considering the relationship between the state and the predicted word, but also considering the image [78]. The relationship between the region and the word and state is more comprehensive.

### 3.11. Deliberate Attention

**Figure 9:**
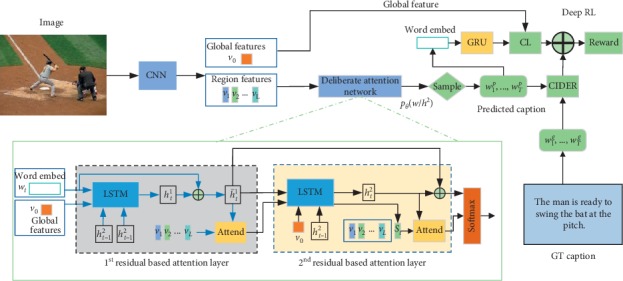
Deliberate attention framework.

Gao et al. [79] proposed a deliberate attention model (Figure 9). The method is proposed by observing people's daily habits of dealing with things, such as a common behavior of improving or perfecting work in people's daily writing, painting, and reading. In the paper, the authors present a novel Deliberate Residual Attention Network, namely DA, for image captioning. The first-pass residual-based attention layer prepares the hidden states and visual attention for generating a preliminary version of the captions, while the second-pass deliberate residual-based attention layer refines them. Since the second-pass is based on the rough global features captured by the hidden layer and visual attention in the first-pass, the DA has the potential to generate better sentences. They also further equip the DA with discriminative loss and reinforcement learning to disambiguate image/caption pairs and reduce exposure bias.

This chapter analyzes the algorithm models of different attention mechanisms.  Table 1 summarizes the application of attention mechanism in image description and points out the comments of different attention mechanisms and the way they add models, which is convenient for readers to choose appropriate in future research. The attention mechanism improves the model's effect.

## 4. Dataset and Evaluation

This chapter mainly introduces the evaluation methods of open-source datasets and generated sentences in this field. Data, computational power, and algorithms are the three major elements of the current development of artificial intelligence. The three complement each other and enhance each other. It can be said that a good dataset can make the algorithm or model more effective. The image description task is similar to machine translation, and its evaluation method extends from machine translation to form its own unique evaluation criteria.

### 4.1. Dataset

**Figure 10:**
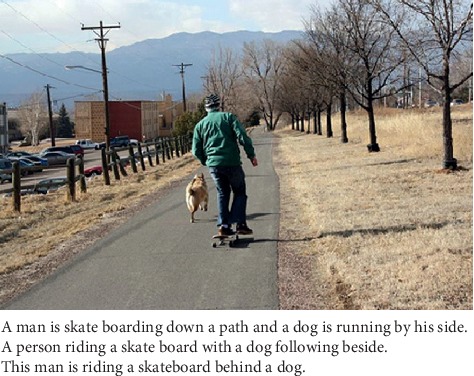
An example in MSCOCO dataset image.

Data are the basis of artificial intelligence. People are increasingly discovering that many laws that are difficult to find can be found from a large amount of data. In the image description generation task, there are currently rich and colorful datasets, such as MSCOCO, Flickr8k, Flickr30k, PASCAL 1K, AI Challenger Dataset, and STAIR Captions, and gradually become a trend of contention. In the dataset, each image has five reference descriptions, and Table 2 summarizes the number of images in each dataset. In order to have multiple independent descriptions of each image, the dataset uses different syntax to describe the same image. As illustrated in the example in Figure 10, different descriptions of the same image focus on different aspects of the scene or are constructed using different grammars.

 
*MSCOCO*. Microsoft COCO Captions dataset [80], developed by the Microsoft Team that targets scene understanding, captures images from complex daily scenes and can be used to perform multiple tasks such as image recognition, segmentation, and description. The dataset uses Amazon's “Mechanical Turk” service to artificially generate at least five sentences for each image, with a total of more than 1.5 million sentences. The training set contains 82,783 images, the validation set has 40,504 images, and the test set has 40,775 images. Its 2014 version of the data has a total of about 20G pictures and about 500M of annotation files which mark the correspondence between one image and its descriptions.
 
*Flickr8k/Flickr30k* [81, 82]. Flickr8k image comes from Yahoo's photo album site Flickr, which contains 8,000 photos, 6000 image training, 1000 image verification, and 1000 image testing. Flickr30k contains 31,783 images collected from the Flickr website, mostly depicting humans participating in an event. The corresponding manual label for each image is still 5 sentences.
 
*PASCAL 1K* [83]. A subset of the famous PASCAL VOC challenge image dataset, which provides a standard image annotation dataset and a standard evaluation system. The PASCAL VOC photo collection consists of 20 categories, and for its 20 categories, 50 images were randomly selected for a total of 1,000 images. Then, Amazon's Turkish robot service is used to manually mark up five descriptions for each image. The dataset image quality is good and the label is complete, which is very suitable for testing algorithm performance.
 
*AIC*. The Chinese image description dataset, derived from the AI Challenger, is the first large Chinese description dataset in the field of image caption generation. The dataset contains 210,000 pictures of training sets and 30,000 pictures of verification sets. Similar to MSCOCO, each picture is accompanied by 5 Chinese descriptions, which highlight important information in the image, covering the main characters, scenes, actions, and other contents. Compared with the English datasets common to similar scientific research tasks, Chinese sentences usually have greater flexibility in syntax and lexicalization, and the challenges of algorithm implementation are also greater.
 
*STAIR*. The Japanese image description dataset [84], which is constructed based on the images of the MSCOCO dataset. STAIR consists of 164,062 pictures and a total of 820,310 Japanese descriptions corresponding to each of the five pictures. It is the largest Japanese image description dataset.

### 4.2. Evaluation Criteria

In the evaluation of sentence generation results, BLEU [85], METEOR [86], ROUGE [87], CIDEr [88], and SPICE [89] are generally used as evaluation indexes. For five indicators, BLEU and METEOR are for machine translations, ROUGE is for automatic summary, and CIDEr and SPICE are present for image caption. They measured the consistency of the n-gram between the generated sentences, which was affected by the significance and rarity of the n-gram. At the same time, all four indicators can be directly calculated by the MSCOCO title assessment tool. The source code is publicly available.

 
*BLEU*. It is the most widely used evaluation indicator; the original intention of the design is not for the image caption problem, but for the machine translation problem based on the accuracy rate evaluation. It is used to analyze the correlation of n-gram between the translation statement to be evaluated and the reference translation statement. Its core idea is that the closer the machine translation statement is to a human professional translation statement, the better the performance. In this task, the processing is the same as machine translation: multiple images are equivalent to multiple source language sentences in the translation. The advantage of BLEU is that the granularity it considers is an n-gram rather than a word, considering longer matching information. The disadvantage of BLEU is that no matter what kind of n-gram is matched, it will be treated the same. For example, the importance of verb matching should be intuitively greater than the article. The higher the BLEU score, the better the performance.
 
*METEOR*. METEOR is also used to evaluate machine translation, which aligns the translation generates from the model with the reference translation and matches the accuracy, recall, and *F*-value of various cases. What makes METEOR special is that it does not want to generate very “broken” translations and the method is based on the precision of one gram and the harmonic mean of the recall. The weight of the recall is a bit higher than the precision. This criterion also has features that are not available in others. It is designed to solve some of the problems with BLEU. It is highly relevant to human judgment and, unlike BLEU, it has a high correlation with human judgment not only at the entire collection but also at the sentence and segment level. The higher the METEOR score, the better the performance.
 
*ROUGE*. ROUGE is a set of automated evaluation criteria designed to evaluate text summarization algorithms. The higher the RUGE score, the better the performance.
 
*CIDEr*. CIDEr is specifically designed for image annotation problems. It measures the consistency of image annotation by performing a Term Frequency-Inverse Document Frequency (TF-IDF) weight calculation for each n-gram. This indicator treats each sentence as a “document,” represents it in the form of a TF-IDF vector, and then calculates the cosine similarity of the reference description to the description generated by the model as a score. In other words, it is the vector space model. This indicator compensates for one of the disadvantages of BLEU, that is, all words on the match are treated the same, but in fact, some words should be more important. Again, the higher the CIDEr score, the better the performance.
 
*SPICE*. It is a semantic evaluation indicator for image caption that measures how image titles effectively recover objects, attributes, and relationships between them. On the natural image caption dataset, SPICE is better able to capture human judgments about the model's subtitles, rather than the existing n-gram metrics.

Table 3 shows the scores of the attention mechanisms introduced in part 3. From Table 3, we found that the scores on different evaluation criteria for different models' performance are not the same. Although there are differences in some evaluation criteria, if the improvement effect of an attention model is very obvious, in general, all evaluation indicators are relatively high for its rating.

Based on the NIC model [49] as state-of-the-art performance, Xu et al. [69] describe approaches to caption generation that attempt to incorporate a form of attention with two variants: a “hard” attention mechanism and a “soft” attention mechanism. Encouraged by recent advances in caption generation and inspired by recent success in employing attention in machine translation [57] and object recognition [90, 91], they investigate models that can attend to a salient part of an image while generating its caption.

Existing approaches are either top-down, which start from a gist of an image and convert it into words, or bottom-up, which come up with words describing various aspects of an image and then combine them. You et al. [89] propose a new algorithm that combines both approaches through a model of semantic attention. The algorithm learns to selectively attend to semantic concept proposals and fuse them into hidden states and outputs of recurrent neural networks. The selection and fusion form a feedback connecting the top-down and bottom-up computation. The method is slightly more effective than the “soft” and “hard” attention.

Visual attention models are generally spatial only. Chen et al. [77] introduce a novel convolutional neural network dubbed SCA-CNN that incorporates spatial and channel-wise attentions in a CNN. In the task of image captioning, SCA-CNN dynamically modulates the sentence generation context in multilayer feature maps, encoding where and what the visual attention is. Pedersoli and Lucas [89] propose “Areas of Attention,” the approach models the dependencies between image regions, caption words, and the state of an RNN language model, using three pairwise interactions, this method allows a direct association between caption words and image regions. Both two methods mentioned above together yield results mentioned earlier on the MSCOCO dataset.

Lu et al. [75] propose a adaptive attention model with a visual sentinel. The model not only decides whether to attend to the image or to the visual sentinel but also decides where, in order to extract meaningful information for sequential word generation. This sets the new state-of-the-art by a significant margin so far.

## 5. Conclusion

In this overview, we have compiled all aspects of the image caption generation task, discussed the model framework proposed in recent years to solve the description task, focused on the algorithmic essence of different attention mechanisms, and summarized how the attention mechanism is applied. We summarize the large datasets and evaluation criteria commonly used in practice.

Although image caption can be applied to image retrieval [92], video caption [93, 94], and video movement [95] and the variety of image caption systems are available today, experimental results show that this task still has better performance systems and improvement. It mainly faces the following three challenges: first, how to generate complete natural language sentences like a human being; second, how to make the generated sentence grammatically correct; and third, how to make the caption semantics as clear as possible and consistent with the given image content. For future work, we propose the following four possible improvements:

1. An image is often rich in content. The model should be able to generate description sentences corresponding to multiple main objects for images with multiple target objects, instead of just describing a single target object.
2. For corpus description languages of different languages, a general image description system capable of handling multiple languages should be developed.
3. Evaluating the result of natural language generation systems is a difficult problem. The best way to evaluate the quality of automatically generated texts is subjective assessment by linguists, which is hard to achieve. In order to improve system performance, the evaluation indicators should be optimized to make them more in line with human experts' assessments.
4. A very real problem is the speed of training, testing, and generating sentences for the model should be optimized to improve performance.

## Figures and Tables

**Table 1:** Comparison of attention mechanism modeling methods.

Ref.	Attention name	Method	Comment
[69]	Soft attention	Give a probability according to the context vector for any word in the input sentence when seeking attention probability distribution	Parameterization Derivative enable Definitely

[69]	Hard attention	Focus only on a randomly chosen location using Monte Carlo sampling to estimate the gradient	Randomly On the base of probability Simple

[70]	Multihead attention	Linearly projecting multiple pieces of information selected from the input in parallel using multiple keys, values, and queries	Linear projection Parallel Focus on information from different representation subspaces in different locations Multiple attention head

[70]	Scaled dot-product attention	Execute a single attention function using keys, values, and query matrices	High speed Save space

[71]	Global attention	Considering the hidden layer state of all encoders, the weight distribution of attention is obtained by comparing the current decoder hidden layer state with the state of each encoder hidden layer	Comprehensive Time-consuming Large amount of calculation

[71]	Local attention	First find a location for it, then calculate the attention weight in the left and right windows of its location, and finally weight the context vector	Reduce the cost of calculations

[75]	Adaptive attention	Define a new adaptive context vector which is modeled as a mixture of the spatially attended image features and the visual sentinel vector. This trades off how much new information the network is considering from the image with what it already knows in the decoder memory	Solve when and where to add attention in order to extract meaningful information for sequence words

[76]	Semantic attention	Select semantic concepts and incorporate them into the hidden state and output of the LSTM	Optional Merge From top to bottom From bottom to top

[77]	Spatial and channel-wise attention	Select semantic attributes based on the needs of the sentence context	Multiple semantics In order to overcome the problem of overrange when using the general attention

[4]	Areas of attention	Modeling the dependencies between image regions, title words, and the state of the RNN language model	Interaction Comprehensive

**Table 2:** Summary of the number of images in each dataset.

Dataset name	Size		
	Train	Valid	Test
MSCOCO	82783	40504	40775
Filckr8k	6000	1000	1000
Filckr30k	28000	1000	1000
PASCAL 1K	—	—	1000
AIC	210000	30000	30000
STAIR	82783	40504	40775

**Table 3:** Scores of attention mechanisms based on the evaluations above.

Ref.	Attention model	BLEU-4	METEOR	ROUGE-L	CIDEr
[69]	Soft attention	24.3	23.9	—	—
[69]	Hard attention	25.0	23.0	51.6	86.5
[70]	Multihead/scaled dot-product	28.4	—	—	—
[71]	Global/local attention	25.9	—	—	—
[75]	Adaptive attention	33.2	26.6	55.0	108.5
[76]	Semantic attention	30.4	24.3	53.5	94.3
[77]	Spatial and channel-wise	31.1	25.4	53.0	94.3
[4]	Areas of attention	31.9	25.2	—	98.1
[79]	Deliberate attention	37.5	28.5	58.2	125.6

## Data Availability

The datasets involved in the paper are all publicly available: MSCOCO [75], Flickr8k/Flickr30k [76, 77], PASCAL [4], AIC AI Challenger website: https://challenger.ai/dataset/caption, and STAIR [78].
